# Emerging Roles of Estrogen-Related Receptors in the Brain: Potential Interactions with Estrogen Signaling

**DOI:** 10.3390/ijms19041091

**Published:** 2018-04-05

**Authors:** Kenji Saito, Huxing Cui

**Affiliations:** 1 Department of Pharmacology, University of Iowa Carver College of Medicine, Iowa City, IA 52242, USA; kenji-saito@uiowa.edu; 2 F.O.E. Diabetes Research Center, University of Iowa Carver College of Medicine, Iowa City, IA 52242, USA; 3 Obesity Research and Education Initiative, University of Iowa Carver College of Medicine, Iowa City, IA 52242, USA

**Keywords:** estrogen, estrogen-related receptors, estrogen receptor, brain, central nervous system, mitochondria

## Abstract

In addition to their well-known role in the female reproductive system, estrogens can act in the brain to regulate a wide range of behaviors and physiological functions in both sexes. Over the past few decades, genetically modified animal models have greatly increased our knowledge about the roles of estrogen receptor (ER) signaling in the brain in behavioral and physiological regulations. However, less attention has been paid to the estrogen-related receptors (ERRs), the members of orphan nuclear receptors whose sequences are homologous to ERs but lack estrogen-binding ability. While endogenous ligands of ERRs remain to be determined, they seemingly share transcriptional targets with ERs and their expression can be directly regulated by ERs through the estrogen-response element embedded within the regulatory region of the genes encoding ERRs. Despite the broad expression of ERRs in the brain, we have just begun to understand the fundamental roles they play at molecular, cellular, and circuit levels. Here, we review recent research advancement in understanding the roles of ERs and ERRs in the brain, with particular emphasis on ERRs, and discuss possible cross-talk between ERs and ERRs in behavioral and physiological regulations.

## 1. Introduction: Estrogen Receptors (ERs) and Estrogen-Related Receptors (ERRs)

Estrogens are steroid hormones known to regulate a wide range of physiological functions, including but not limited to reproduction, cardiovascular physiology, homeostatic regulation of energy balance, and a variety of social and learning behaviors. Traditionally, the actions of circulating estrogen were believed to be mediated mainly by binding to two specific receptors, estrogen receptors α (ERα) and estrogen receptors β (ERβ), which recognize and activate gene transcription through binding to the genomic element called the estrogen-response element (ERE), either as a homodimer or heterodimer with coactivators [1,2]. Notably, apart from their well-known roles in transcriptional regulation, estrogens were also recently reported to rapidly activate extracellular signal-regulated kinases (ERKs) according to a new mode of action of ERs as well as the expression of an orphan G-protein-coupled receptor 30 (GPR30), that functions as a novel type of ER. As such, even after nearly a century since their discovery, the exact mechanisms by which estrogens regulate different physiological functions are still incompletely understood and remain an active area of research.

The estrogen-related receptors α and β (ERRα and ERRβ) were the two first orphan nuclear receptors identified based on their sequence similarity to the ERα [3]. Together with ERRγ, these three receptors consist of the ERR subfamily of the group III steroid nuclear receptor superfamily. Other group III nuclear receptors include glucocorticoid, mineralocorticoid, progesterone, and androgen receptors as well as ERs. Although ERRs share sequence homologies with ERs, estrogens are not their natural ligands and ERRs exhibit constitutive activity and can work as transcriptional regulators in the absence of ligands [4]. The ERRs contain DNA-binding domains (DBDs) constituting two highly conserved zinc finger motifs that target the receptor to a specific DNA sequence (TCAAGGTCA) called the estrogen-related response element (ERRE). ERRs bind to ERRE as a monomer or a homodimer or as a heterodimer with co-activators [5,6]. In addition to ERRE, ERRs can also bind to ERE and, conversely, ERα, but not ERβ, and can bind to ERRE as well [7], implying shared transcriptional networks driven by both ERRs and ERα. Not surprisingly, in many tissues both ERα and ERRs are highly expressed, including metabolically active skeletal muscle, fat and brain [8,9], but whether and how they are coordinated to control shared and/or distinct transcriptional events remain unclear. Compared to ERs, our knowledge about the tissue- and cell type-specific roles of ERRs are limited. Further studies are needed to uncover transcriptional networks driven by ERRs in different cell types and to investigate how they will affect whole-body physiology either independently or in coordination with estrogen signaling.

## 2. ERs and Their Modes of Action

As classical nuclear receptors, upon ligand binding ERs translocate to the nucleus and are directly recruited to the EREs on the target genes. This mode of action is called the genomic action of estrogens. However, as mentioned, the estrogen signals can also be mediated through rapid, cytosolic ER-initiated signaling cascades. Mutant female mice in which ERα’s ability to bind to the EREs was disrupted, are infertile and display a variety of abnormalities in the reproductive system [10]. However, this mutation in ERα null background restores the obese phenotype of ERα knockout mice [11], indicating that ERα’s role in the homeostatic regulation of the energy balance is independent of its genomic action. One likely signaling pathway downstream of ERα to exert its rapid, membrane-initiated action is PI3K/Akt pathway. Estradiol activates the PI3K/Akt pathway in hypothalamic nuclei [11,12,13]. Genetic inactivation of the PI3K pathway in hypothalamic nuclei blunts the anti-obese effects of estrogens [14,15]. Although the involvement of classical genomic ERα signaling cannot be fully ruled out, these studies suggest a critical role of rapid, membrane-initiated actions of ERα on energy homeostasis. Thus, it is plausible that different modes of action of estrogen can exert different physiological functions. The latest member of estrogen receptors, GPR30 (also known as GPER), is a G protein-coupled estrogen receptor that was cloned by several groups in the 1990s [16,17,18,19]. Later, this was followed by numerous reports showing GPR30-dependent 17β-estradiol signaling and actual binding of 17β-estradiol with GPR30 [20,21,22,23,24,25,26,27]. So far, it is unknown whether ERRs can also activate rapid, membrane-initiated signaling pathways as seen in ERs.

## 3. ERRs and Their Potential Ligands

Although ERRs were identified based on their sequence similarities to ERs, estrogen is not a natural ligand of ERRs and to date, endogenous ligand(s) of ERRs remain unclear. Given the established roles of ERRs in energy metabolism and the development of certain types of cancers [28,29,30,31,32], there have been active and continuous efforts to identify their endogenous ligands, transcriptional co-activators, and the synthetic compounds that can be used to modulate the activity of ERRs. Interestingly, a recent study using affinity chromatography of tissue lipidomes with the ERRα ligand-binding domain identified cholesterol as an endogenous ERRα agonist [33]. While this study represents a first successful screening of potential endogenous ligands for ERRs, the specific transcriptional dynamics as well as physiological functions of cholesterol binding to ERRα remain to be fully determined.

Although endogenous ligands of ERRs remain uncertain, several transcriptional co-activators interacting with ERRs have been identified, which include peroxisome proliferator-activated receptor gamma coactivator 1-alpha (PGC1-α) and PGC1-β [4,28,31]. Both PGC1-α and PGC1-β function as protein ligands for ERRs and the transcriptional activities driven by these interactions have been shown to be essential for mitochondrial biogenesis and, thus, cellular energy metabolism [4,34,35]. Therefore, targeting these transcriptional networks may hold promise for treating the metabolic disorders including diabetes, obesity and cancer [8,34,36,37,38].

Additionally, multiple inverse agonists or antagonists have been developed. Synthetic estrogen diethylstilbestrol acts as an inverse agonist for all three ERRs [29]. More selective inverse agonists, such as XCT 790 and DY 131, have been developed for ERRα, ERRβ/γ, respectively [39]. Most of these inverse agonists were designed to block the interactions with their protein-binding partners. XCT 790 blocks the interaction between ERRα and PGC1-α and has been shown to inhibit the expression of ERRα target genes [40] and cancer cell proliferation [41]. Other inverse agonists of ERRα are compound 29 and 50 developed by Janssen Pharmaceuticals, which show high selectivity for ERRα and have strong therapeutic potential for treating obesity and diabetes [38]. The estrogenic chemical Bisphenol A has also been reported to be a potential agonist for ERRγ [42]. Additionally, there is some evidence showing that dietary products, such as resveratrol, genistein, rutacarpine, piceatanol and flavone, could function as potential agonists of ERRα [43,44]. More recently, a study screening the Tox21 compound library has identified multiple potential novel ERR agonists, including a potent histone deacetylase inhibitor Suberoylanilide hydroxamic acid (SAHA) and a class of lipid-lowering medication statins, including atorvastatin, fluvastatin, and lovastatin [45]. As such, the use of these natural and synthesized ligands will facilitate the processes of studying the whole-body effects of modulating the transcriptional activities of ERRs and help to develop an effective therapeutic strategy for the diseases associated with ERRs, including cancers and metabolic diseases such as obesity and diabetes. 

## 4. Transcriptional Regulations by Both ERs and ERRs

ER-signaling is involved in the development of breast cancer. Three quarters of breast tumors are considered to express ERα [46,47]. The estrogen signaling through ERα regulates the expression of various genes that play key roles in cell proliferation and cell-cycle progression [48,49]. As a primary treatment for breast cancer, ER-positive breast cancers are preferentially treated with reagents that suppress ERα signaling such as tamoxifen and aromatase inhibitors. Unfortunately, after years of treatment, the recurrence of breast cancer could happen with a resistance to estrogen-signaling inhibitors. Recently, ERRs have been attracting much attention for the prognosis of ER-positive and negative breast cancer. ERRα expression is high in breast cancer, especially in cancer cells lacking ERα, and is considered as a negative prognostic factor for breast-cancer survival [50,51].

**Figure 1:**
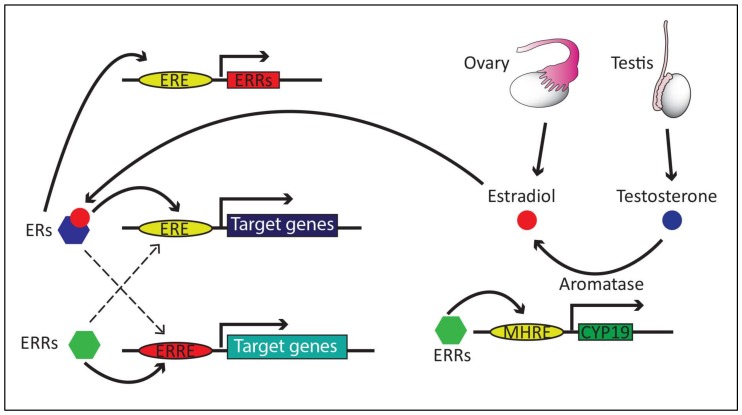
Schematic drawing showing potential cross-talk between estrogen, estrogen receptors (ERs), and estrogen-related receptors (ERRs). Dotted lines indicate relatively weak binding ability. MHRE, multiple-hormone response element; CYP19, aromatase.

While estrogen is not an endogenous ligand of ERRs, there is possible cross-talk between estrogen signaling and ERRs in different ways (Figure 1). Studies showed that the ERRα promoter has multiple steroid hormone response-element half-sites, where ERα could bind, and estrogens stimulate ERRα expression in vivo and in vitro [52,53], suggesting that ERRα is one of the transcriptional targets of ERα. Chromatin immunoprecipitation for ERα and ERRα coupled with microarray revealed that some targeted genes are shared by these two receptors [54]. Both ERα and ERRα stimulate the transcription of Runx2-I, a master regulator of bone development, through a common ERE, and this transcriptional regulation by ERRα is changed dependent on its binding partner; the binding with PGC1-α acts as a transcriptional activator, while the binding with PGC1-β acts as a transcriptional repressor [55]. These studies suggest that ERs and ERRs can cross-talk and mutually regulate the expression of common target genes. However, chromatin immunoprecipitation (ChIP) study also indicates that the occupancy of the shared targets by both ERα and ERRα is relatively modest among each of their transcriptional target genes and, therefore, it is likely that, depending on tissue, both ERα and ERRα maintain a high degree of independence for their transcriptional regulation [54].

Possible interaction between estrogen signaling and ERRs is also supported by a study showing that ERRα regulates the expression of aromatase, an enzyme responsible for the conversion of testosterone to estrogen [56,57]. Aromatase expression is also regulated by ERα [58]. Additionally, it was shown that ERRβ could directly bind to ERα in order to restrain ERα morbidity and suppress estrogen-dependent cellular function [59]. These reciprocal interactions between estrogen, ERs and ERRs warrant future research to investigate (1) whether the levels of ERRα expression changes by ovariectomy or along with different stages of the estrus cycle; (2) whether ERα knockout mice have altered levels of ERRα expression across the tissues; and (3) conversely, do ERRα knockout mice have impaired estrogen signaling and/or reproductive dysfunctions? 

## 5. Functions of ERs and ERRs in the Brain

### 5.1. Actions in the Central Regulation of Energy Homeostasis

Estrogen signaling has been well known to play an essential role in body-weight regulation [60]. Postmenopausal women experience a remarkable decline in circulating 17β-estradiol (E2), which is often associated with the development or accumulation of body fat, obesity, type II diabetes, hypertension, and the metabolic syndrome [61]. The involvement of estrogens in energy homeostasis is more obvious in experimental animal models. The withdrawal of endogenous estrogens by ovariectomy in female animals leads to hyperadiposity and body-weight gain, and this obese phenotype can be prevented by E2 supplementation [62,63]. Conversely, microinjections of E2 into various brain regions suppress feeding behavior and body-weight gain [64,65]. The importance of central estrogen signaling in energy homeostasis was later confirmed by genetic mouse models. Among three cloned estrogen receptors, the estrogenic effects on energy homeostasis are believed to be primarily mediated by ERα. ERα is widely expressed throughout the brain including, but not limited to, those hypothalamic and brainstem nuclei that are important for the homeostatic regulation of energy balance, such as the ventromedial nucleus of the hypothalamus (VMH), the arcuate nucleus of the hypothalamus (ARC), the medial amygdala (MeA), and the nucleus of the solitary tract (NTS). Humans with a mutation in ERα and mice lacking ERα throughout the body or specifically in the brain are obese due to both hyperphagia and/or reduced physical activity and energy expenditure [66,67,68]. Furthermore, the anti-obesity effects of E2 replacement in ovariectomized mice are blocked in ERα knockout mice [62].

More specifically, the knockdown of ERα in the VMH by shRNA blunts E2-mediated weight loss and leads to obesity associated with increased visceral fat [69], likely due to decreased physical activity and impaired thermogenesis, but not food intake. Consistent with these findings, the mice with VMH-specific ERα knockout [68] showed modest weight gain due to reduced energy expenditure but not food intake, and were infertile. 

ERα is also abundantly expressed in the ARC [69]. The ARC contains two primarily distinct but intermingled neuronal populations that express either anorexigenic pro-opiomelanocortin (POMC) or orexigenic agouti-related peptide (AgRP) and neuropeptide Y (NPY). ERα is expressed in POMC neurons [68,70,71] and POMC levels change in response to estrogens [72]. Genetic mouse study revealed that conditional deletion of ERα in POMC neurons leads to hyperphasia and modest weight gain [68]. AgRP/NPY neurons are also modulated by estrogen signaling. Dhillon and Belsham have shown that estrogens inhibit NPY release in immortalized hypothalamic cells through a ERα-dependent mechanism [73]. AgRP and NPY expressions fluctuate along with the estrus stages and the anorexigenic effect of 17β-estradiol was blunted in mice with the ablation of AgRP neurons [74]. NPY neuronal excitability is also modulated by estrogen via a change in K^+^ channel expression [75]. However, which type of estrogen receptors are responsible for estrogen effects on AgRP/NPY neurons is controversial. Immunohistochemistry failed to detect ERα in NPY neurons despite clear estrogen effects on this neuronal population [74]. On the other hand, others reported colocalization of NPY and ERα [76,77].

ERα is also highly expressed in the medial amygdala (MeA). Conditional deletion of ERα in the MeA led to weight gain in both male and female mice, mainly due to decreased energy expenditure associated with low physical activity, but not food intake [78]. Interestingly, both male and female aromatase knockout mice develop obesity due to lowered physical activity, and this body weight gain is not associated with hyperphasia [79], resembling phenotypes observed in mice lacking ERα in MeA.

**Figure 2:**
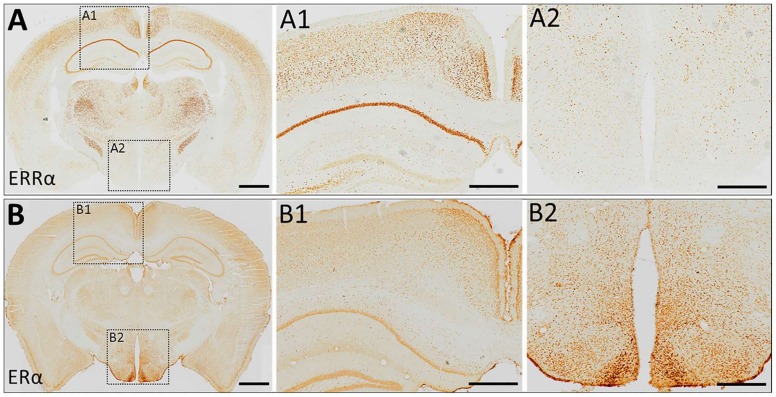
Representative immunohistochemistry (IHC) images showing ERRα (**A**) and ERα (**B**) expression in the mouse brain. Digital zooms of individual boxed regions are shown in A1, A2, B1, and B2. Note that expression pattern of ERRα and ERα is similar in the cortex and the hippocampus (**A1**,**B1**), but strikingly different expression was observed in the mediobasal hypothalamus (**A2**,**B2**) that is critical for the homeostatic regulation of energy balance. Note: the IHC image shown in (**A**) was from our previous publication with a zoom-in view [81]. The IHC image shown in (**B**) was from the brain sections of an adult female wild-type mouse stained with validated commercially available ERα antibody (1:1000, Millipore) as reported previously [71]. This ERα antibody was validated in conditional ERα KO mice previously [68]. Scale bar = 1 mm in A and B, and 500 µm in A1, A2, B1, and B2.

As such, ERα expressed by distinct types of neurons in the brain seemingly plays differential roles in maintaining whole-body energy homeostasis as reviewed elsewhere [60]. By contrast with the positive energy balance observed in ERα knockout mice, however, it has been reported that conventional ERRα knockout mice have reduced body weight and fat mass compared to their control littermates, especially when challenged with high fat diet (HFD) [80]. While food intake seemed comparable between knockout and control groups in an early report [80], we have recently found a significant reduction of palatable HFD intake in ERRα knockout mice that is associated with a significant reduction of body weight [81]. This is not only with general consumption of HFD, and we also found that hungry ERRα knockout mice display less willingness to obtain HFD pellets in an operant-responding behavioral paradigm with a progressive ratio schedule compared to their control littermates, suggesting a reduced motivation to work for palatable food [81]. Interestingly, through a family-based genetic linkage study combined with whole exome sequencing in a family in which multiple members are affected by eating disorders, particularly anorexia nervosa, we have identified a missense mutation in the ERRα gene that co-segregates with illness [82]. A subsequent study in mice revealed that the level of expression of ERRα in the brain is increased by caloric restriction, implying that brain ERRα may sense peripheral energy status and convert it into protective behavioral actions, including motivation to obtain and consume food [81]. It is possible that a genetic deficit of ERRα disrupts this adaptive (protective?) physiological process upon caloric restriction, leading to pathological conditions such as eating disorders [81]. Somewhat consistent with the differential role of ERRα and ERα in body-weight homeostasis, the expression pattern of ERRα in the hypothalamus is different from ERα. Unlike the high expression of ERα in the mediobasal hypothalamus, which is critical for the coordinated control of energy balance [68], ERRα expression is nearly absent in the mediobasal hypothalamus (Figure 2). However, it should also be noted that other than the mediobasal hypothalamus, in many brain regions, including the cortex and hippocampus, both ERα and ERRα are homogeneously expressed (Figure 2). From the viewpoint of brain reward circuits [83,84], however, some of the extra-hypothalamic regions, such as several frontal cortices and the hippocampus, express relatively high levels of ERRα, and the ventral striatum, ventral pallidum, and lateral hypothalamus with moderate levels [81]. Interestingly, the ventral tegmental area (VTA), a key brain region of brain reward function, had minimal expression of ERRα [81]. We have previously shown that specific knockdown of ERRα in the medial prefrontal cortex (mPFC) recapitulates reduced motivation for HFD observed in Esrra-null female mice, indicating that Esrra expression in the mPFC may affects top-down control of food reward behaviors [81]. Nonetheless, it is possible that ERRα expression in the periphery, rather than the brain, is responsible for protected weight gain observed in ERRα knockout mice on HFD feeding. Further studies with conditional a ERRα deletion approach are necessary to prove this possibility.

ERRγ knockout mice die shortly after birth due to cardiac failure [85] and, therefore, the role of ERRγ in the regulation of body weight homeostasis remains elusive. A recent study with exclusive overexpression of ERRγ in skeletal muscle of obese db/db mice revealed that gain of function of ERRγ in skeletal muscle does not ameliorate obesity or diabetic phenotypes in leptin receptor deficiency [34]. ERRγ is also abundantly expressed throughout the brain [86,87], and colocalization of ERRγ and ERα is confirmed within the same neurons in selected regions [86]. Again, studies with conditional ERRγ deletion models are necessary to determine the effects of ERRγ in the long-term regulation of energy homeostasis.

Like ERRγ knockout, global knockout of ERRβ is also lethal for mice [88]. However, a recent study with conditional deletion of ERRβ using Sox2-Cre revealed that these mice are viable and exhibit significantly decreased body weight compared to their littermate controls mainly due to increased activity and energy expenditure [89]. In fact, food intake was significantly increased, which is likely a compensatory response for extremely increased energy expenditure. Paradoxically, however, when ERRβ is deleted from the central nervous system using nestin-Cre, these mice exhibit significantly increased body weight while maintaining higher activity and energy expenditure. Consistent with these results, it was found that increased body weight was mainly due to increased lean mass, but not fat mass, explaining their increased energy expenditure. Interestingly, a loss of ERRβ caused significant upregulation of ERRγ which, the author concluded, might be responsible for the decreased NPY expression in these mice affecting the satiety response [89]. Thus, the ERRβ is clearly involved in long-term regulation of the energy balance, but underlying mechanisms seem complicated, involving a combination of changes of food intake, meal pattern, activity, and energy expenditure by different mechanisms.

Compared to peripheral tissues, the study of the cross-talk between ERs and ERRs in the brain is limited. Further investigation is needed to clarify their cellular colocalization and to better understand the coordinated actions of these relatives. 

### 5.2. Actions in Learning and Memory

Estrogens are known to affect the hippocampus, a large brain structure critical for learning and memory. Estrogen can acutely modulate the electrophysiological properties of hippocampal neurons in ex vivo slice preparations [90,91,92]. Since this effect is rapid, estrogen is thought to work through a rapid, membrane-initiated mechanism in this regard. Membrane-initiated estrogen-signaling activates various protein kinase cascades such as PI3K, protein kinase A, protein kinase C, phospho lipase C, and mitogen-activated protein kinase, leading to the modulation of signal transduction, protein phosphorylation, and ion channel activity [76]. Two estrogen receptors, ERα and ERβ, appear to be located predominantly in synapses, axons, dendrites and dendritic spines [93,94], and work differentially in the hippocampal inhibitory and excitatory synapses, respectively. Estradiol acutely enhances excitatory postsynaptic currents, which can be recapitulated by ERβ-specific agonist diaryl-propionitrile (DPN), but not ERα-specific agonist propyl-pyrazole triol (PPT) [95]. On the other hand, estradiol works through ERα to suppress inhibitory neurotransmission in the hippocampal CA1 neurons [96]. Estrogen treatment rapidly increases dendritic spine density in CA1 of the hippocampus associated with improved spatial learning and memory [97]. Rapid estrogen signaling is observed even with E2-BSA, a membrane-impermeable conjugate of estrogen [98,99]. In addition to these classical ERs, a number of studies using different technologies indicate that rapid estrogen signaling mediated by receptors other than ERα/β exists. E2 can potentiate kainate-induced currents, which can be observed even in the hippocampus of ERα KO mice. In addition, this E2-induced potentiation is unaffected even in the presence of ERα/β blocker [100]. Selective ligand to G-protein coupled estrogen receptor STX, that does not bind to ERα and ERβ, and activates G-protein signaling cascade [99].

Despite considerably high expression of ERRs, especially ERRα and ERRγ, in hippocampal formation, their roles in memory and learning are largely unexplored. Some evidence suggests that ERRγ-mediated gene transcriptions may affect hippocampal functions. It was shown that bisphenol A, a potential agonist for ERRγ and ERα/β, modulates spinogenesis in adult hippocampal neurons through ERRγ, but not ERα/β. Pei et al. showed that ERRγ-deficient hippocampal neurons exhibit lower metabolic capacity [101]. In addition, ERRγ-deficient hippocampal slices showed a significant reduction in long-term potentiation (LTP), which could be rescued by pyruvate supplementation, indicating that the impaired LTP is likely caused by the metabolic deficiency. Consistent with these observations, ERRγ knockout mice showed impaired spatial learning and memory [101]. Although ERRα is also widely expressed throughout hippocampal formation (Figure 2), its role in learning and memory remains to be explored. We have previously shown that the ability of learning and memory in ERRα knockout mice was comparable to wild-type littermates in a Barnes maze test, but reversible learning in this behavioral paradigm was significantly impaired in ERRα knockout mice [81]. However, impaired reversible learning in a Barnes maze test could be interpreted as an indication of behavioral rigidity, rather than memory per se. Interestingly, Wrann et al. have recently shown that exercise-induced increases of brain-derived neurotrophic factor (BDNF), a neurotrophic factor essential for synaptic plasticity, hippocampal function and learning, is mediated by elevated expression levels of fibronectin type III domain containing 5 (FNDC5) (precursor of a novel, circulating myokine irisin) that is driven by a ERRα/PGC1α transcriptional network [40]. These findings indicate that, while the ERRα may plays a minimal role in learning under normal physiological conditions, it might be an important molecular mediator of exercise-induced beneficial effects in improving learning and memory through the ERRα/PGC1α→FNDC5→BDNF pathway. Indeed, it has been postulated that, rather than in a baseline condition, the roles ERRα plays become more apparent when animals are subjected to various physiological and environmental challenges requiring them to make adaptive responses [102]. Therefore, the potential role of ERRα in learning and memory needs to be further investigated under different physiological conditions or by different behavioral learning and memory tasks. Additionally, we have previously found that both presynaptic vesicle pool density and the numbers of dendritic spines are significantly decreased in the striatum of female ERRα knockout mice [103]. Although it remains unclear whether similar changes also occur in the hippocampus, these findings are suggestive of impaired synaptic plasticity in ERRα knockout mice by impaired synaptic vesicle trafficking and/or synaptogenesis. It will be interesting to test whether exercise-induced beneficial effects on improving learning and memory are lost or impaired in ERRα-deficient mice.

### 5.3. Actions in Social Behaviors

Estrogens have been shown to impact a wide range of social behaviors, and ERα and ERβ may play differential roles in this regard in a gender-specific manner [104]. Global ERα knockout male mice are less aggressive [105], while ERβ knockout mice show enhanced aggressive behavior [106], indicating that ERα is essential for expressing aggressive behavior whereas ERβ works on it in an antagonistic manner. Furthermore, ERα in distinct brain nuclei differentially regulates male aggressive and sexual behaviors. Adeno-associated virus (AAV)-mediated ERα knockdown in the medial preoptic area reduced sexual but not aggressive behavior while the knockdown in the VMH suppressed both behaviors. On the other hand, the knockdown in the MeA had no effects on either behavior [107]. MeA also expresses aromatase. Selective ablation of aromatase-expressing cells in the MeA suppresses male aggressive behavior and female maternal aggressive behavior [108].

The involvement of estrogen-signaling in social behaviors should not ignore its function in the perceptions of others [109]. Ovariectomized female animals are less attractive to males than intact female animals, which can be restored by estrogen supplementation [110,111]. Genetic studies suggest that odors produced from either ERα knockout or ERβ knockout female mice are different from those from wild-type female mice, and wild-type male mice show less interest in ERα knockout or ERβ knockout female mice compared to wild-type female mice [112]. The same thing can be said of the perception of ERα and ERβ knockout male mice by wild-type female mice. Wild-type female mice can successfully discriminate the odors of wild-type males from those of knockout males and display significantly higher preference for the WT male odors.

Classic lesion and electrical stimulation studies have identified the brain loci involved in aggression and other social behaviors, which include the anteroventral periventricular nucleus, the medial preoptic area, the bed nucleus of stria terminalis, and VMH. Additionally, the recent development of optogenetic and pharmacogenetic techniques also greatly facilitated the process of delineating brain circuits that might mediate estrogen actions in these behavioral regulations [99]. The VMH is one of the important nuclei that mediate estrogenic actions in energy homeostasis. In addition to energy homeostasis, recent studies using optogenetic and pharmacogenetic tools have begun to unravel the importance of ERα-expressing neurons in the VMH in social behaviors, such as sexual behavior and aggression. Optogenetic and pharmacogenetic activation of ERα-expressing neurons in the VMH trigger aggressive behaviors in both males and females [113,114]. This ERα-expressing subpopulation of VMH neurons seem to not only control aggressive behavior but also involve other social behaviors such as investigation and mounting in males, and increasing both the number of active neurons and the activity level of each neuron can shift the behavioral responses from mounting to attacking [113]. A separate study targeting ERα-expressing neurons with same Cre mouse model also showed that VMH ERα-positive neurons are highly active during attacking and are necessary for female aggressive behavior as well [114]. These studies clearly showed the involvement of ERα-expressing neurons in aggressive behavior, but it remains unclear whether signaling through ERs itself is important for the aggressive behaviors.

ERRs, especially ERRα, also regulate social behaviors. Impaired social function was one of the characteristic behavioral deficits observed in ERRα knockout mice [81]. ERRα knockout mice showed reduced interaction with a novel mouse tested in a social interaction test. Furthermore, in the tube test which measures the dominance tendency, ERRα knockout mice were almost always the losers [81]. The recent sophisticated behavioral and optogenetic studies showed that the activity of the dorsomedial prefrontal cortex (dmPFC), where ERRα is highly expressed, is important for instant winning or losing in the tube test [115,116]. It will be interesting to test if optogenetic activation of dmPFC ERRα-positive neurons can rescue social subordination seen in ERRα knockout mice. It is difficult to associate the VMH-regulated aggressive behavior with dmPFC-regulated social dominance. Social dominance is important for survival in social animals and aggression could be a means to reach the top of the social hierarchy. It is noteworthy that the selective knockdown of ERRα in distinct subnuclei of the PFC can recapitulate some behavioral deficits observed in ERRα knockout mice, such as reduced body weight and food intake, reduced effort responding for food reward, and increased grooming [81]. Recently, synchronized activity among distant brain nuclei (PFC-lateral septum-lateral hypothalamus) is suggested to be involved in food-seeking behavior [117]. Whether such a synchronicity exists between PFC and VMH for the coordinated control of social dominance and/or aggressive behavior remains to be determined. Cortical parvalbumin (PV)-expressing inhibitory interneurons are a potent regulator of local network activities [118] and coherent activity of PV neurons orchestrates synchronous gamma oscillations [119,120]. Widely distributed expression patterns of ERRs (both ERRα [81] and ERRγ [86,87]) throughout the brain are indicative of their likely expression in both excitatory and inhibitory neurons. Future studies of conditional deletion models are required to distinguish their roles in different types of neurons in behavioral regulations. Given an established role of ERRs in mitochondrial biogenesis [121,122] and, thus, cellular energy supply, it would be interesting to investigate whether ERRs are enriched in cortical inhibitory interneurons with high energy demand, particularly fast-spiking PV neurons [123,124], and whether loss of ERRs will cause aberrant firing of these neurons due to insufficient cellular energy production, leading to behavioral abnormalities including social behaviors. In line with the involvement of ERRs in mitochondrial biogenesis in GABAergic interneurons, its protein ligand PGC1-α, a master regulator of mitochondrial biogenesis, is predominantly expressed in these neuronal populations [125] and bidirectionally regulates PV expression [126]. PGC1-α in PV neurons transcriptionally regulates genes relevant to synaptic transmission, as well as metabolism-related genes and conditional deletion of PGC1-α in PV neurons results in asynchronous GABA release and impaired long-term memory [127]. Notably, a recent comprehensive gene expression profiling in the hypothalamus, frontal cortex, and amygdala by RNA-Seq combined with ChIP-Seq revealed that ERRα is in the center of coordinating transcriptional networks for adaptive responses when animals are challenged to agonistic social encounters [128]. Transcriptional regulatory dynamics that take place under social challenges could be essential for animals to learn from this type of social challenge and affect future behaviors critical for survival. The roles of ERRα in adaptive metabolic and behavioral responses to social stressors warrant future investigation. Additionally, a variety of behavioral deficits caused by ERRα deletion are sexually dimorphic [81], yet no sexually dimorphic expression patterns of ERRα in the brain were observed. Further studies are needed to clarify the underlying mechanisms of sexual dimorphic roles of ERRα in these behavioral regulations.

## 6. Concluding Remark

**Figure 3:**
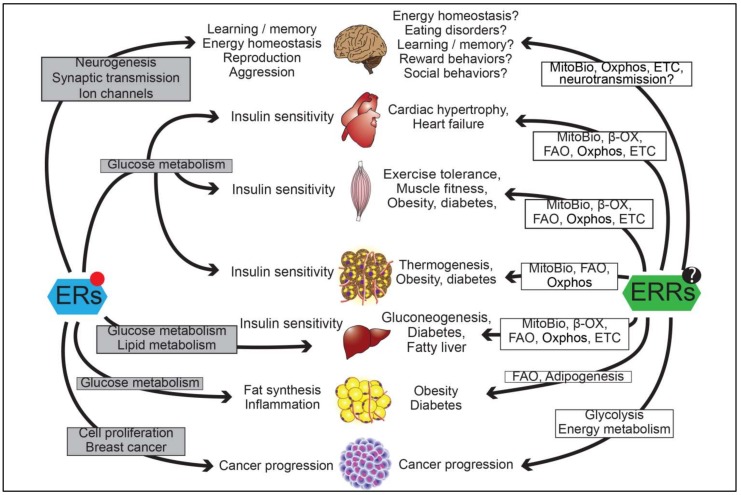
ERs and ERRs regulate a variety of important cellular functions and any disruption in these processes can lead to different pathological conditions. MitoBio, mitochondrial biogenesis; β-OX, beta-oxidation; FAO, fatty acid oxidation; Oxphos, oxidative phosphorylation; ETC, electron transport chain. Red dot indicates estrogens and “?” indicates unknown ligands for ERRs.

It has been well known that both ERs and ERRs play important roles in physiological regulations through their abundant expression in peripheral tissues, particularly for metabolic homeostasis and energy metabolism (Figure 3). Mounting evidence indicates that the brain is also one of the primary targets of estrogen (via ERs) to regulate a variety of behaviors and physiological functions including reproduction, energy homeostasis, and learning and memory. ERRs share sequence similarity with ERs, but estrogen is not their endogenous ligand and little attention has been paid to the cross-talk between estrogen-signaling and ERRs. Existing evidence supports the idea that estrogen-signaling and ERRs may cross-talk via transcriptional regulation, or reciprocal binding on each responsive element, or even intercellularly through the regulation of estrogen synthesis by aromatase. However, the roles of ERRs in the brain and functional segregation of the isoforms remain largely unknown. Additionally, the functional overlaps between ERs and ERRs are almost untouched at the behavior level. Gene-expression profiling studies in peripheral tissues and cell lines indicate that shared target genes by both receptor families may be modest, with a high degree of independence. While the expression patterns of ERα and ERRα suggest that these two receptors might colocalize in some brain regions, to what extent, if any, they share transcriptional targets in the brain is unclear. It is obvious that both families are involved in the processes important for brain functions such as synaptic transmission, neuronal firing and mitochondrial biogenesis. A more comprehensive understanding of the target genes and the transcriptional cross-talk between these receptors may provide more insights into the estrogen-dependent and -independent regulation of brain functions.

## Abbreviations

AgRP	Agouti-Related Peptide
Akt	Protein Kinase B
ARC	Arcuate nucleus Of Hypothalamus
BDNF	Brain-Derived Neurotrophic Factor
β-OX	β-Oxidation
ChIP	Chromatin Immunoprecipitation
CYP19	Aromatase
DBD	DNA Binding Domain
dmPFC	Dorsomedial Prefrontal Cortex
DPN	Diaryl-Propionitrile
E2	17β-Estradiol
ER	Estrogen Receptor
ERE	Estrogen Response Element
ERR	Estrogen-Related Receptor
ERRE	Estrogen-Related Response Element
ERK	Extracellular Signal-Regulated Kinase
ETC	Electron Transport Chain
FAO	Fatty Acid Oxidation
FNDC5	Fibronectin Type III Domain Containing 5
GPER	G protein-Coupled Estrogen Receptor
HFD	High Fat Diet
IHC	Immunohistochemistry
LTP	Long-Term Potenciation
MeA	Medial Amygdala
MHRE	Multiple-Hormone Response element
MitoBio	Mitochondrial Biogenesis
NPY	Neuropeptide
NTS	Nucleus of Solitary Tract
Oxphos	Oxidative Phosphorylation
PV	Parvalbumin
PGC	Proliferator-Activated Receptor γ Coactivator
PI3K	Phosphatidyl Inositide 3-Kinase
POMC	Pro-Opiomelanocortin
PPT	Propyl-Pyrazole Triol
VMH	Ventromedial Nucleus of Hypothalamus
VTA	Ventral Tegmental Area
